# Systematic review and meta-analysis: rapid diagnostic tests *versus *placental histology, microscopy and PCR for malaria in pregnant women

**DOI:** 10.1186/1475-2875-10-321

**Published:** 2011-10-28

**Authors:** Johanna H Kattenberg, Eleanor A Ochodo, Kimberly R Boer, Henk DFH Schallig, Petra F Mens, Mariska MG Leeflang

**Affiliations:** 1 Royal Tropical Institute/Koninklijk Instituut voor de Tropen (KIT), KIT Biomedical Research, Parasitology Unit, Meibergdreef 39, 1105 AZ Amsterdam, the Netherlands; 2 Academic Medical Centre, Centre for Infection and Immunity, Meibergdreef 9, 1105 AZ, Amsterdam, the Netherlands; 3 Academic Medical Centre, Department of Clinical Epidemiology, Biostatistics and Bioinformatics, Meibergdreef 9, 1105 AZ, Amsterdam, the Netherlands; 4 Royal Tropical Institute/Koninklijk Instituut voor de Tropen (KIT), KIT Biomedical Research, Epidemiology Unit, Meibergdreef 39, 1105 AZ Amsterdam, the Netherlands

**Keywords:** Malaria, pregnancy, malaria in pregnancy (MiP), rapid diagnostic tests (RDTs), PCR, microscopy, histology, diagnostic test accuracy, systematic review, meta-analysis

## Abstract

**Background:**

During pregnancy, malaria infection with *Plasmodium falciparum *or *Plasmodium vivax *is related to adverse maternal health and poor birth outcomes. Diagnosis of malaria, during pregnancy, is complicated by the absence or low parasite densities in peripheral blood. Diagnostic methods, other than microscopy, are needed for detection of placental malaria. Therefore, the diagnostic accuracy of rapid diagnostic tests (RDTs), detecting antigen, and molecular techniques (PCR), detecting DNA, for the diagnosis of *Plasmodium *infections in pregnancy was systematically reviewed.

**Methods:**

MEDLINE, EMBASE and Web of Science were searched for studies assessing the diagnostic accuracy of RDTs, PCR, microscopy of peripheral and placental blood and placental histology for the detection of malaria infection (all species) in pregnant women.

**Results:**

The results of 49 studies were analysed in metandi (Stata), of which the majority described *P. falciparum *infections. Although both placental and peripheral blood microscopy cannot reliably replace histology as a reference standard for placental *P. falciparum *infection, many studies compared RDTs and PCR to these tests. The proportion of microscopy positives in placental blood (sensitivity) detected by peripheral blood microscopy, RDTs and PCR are respectively 72% [95% CI 62-80], 81% [95% CI 55-93] and 94% [95% CI 86-98]. The proportion of placental blood microscopy negative women that were negative in peripheral blood microscopy, RDTs and PCR (specificity) are 98% [95% CI 95-99], 94% [95% CI 76-99] and 77% [95% CI 71-82]. Based on the current data, it was not possible to determine if the false positives in RDTs and PCR are caused by sequestered parasites in the placenta that are not detected by placental microscopy.

**Conclusion:**

The findings suggest that RDTs and PCR may have good performance characteristics to serve as alternatives for the diagnosis of malaria in pregnancy, besides any other limitations and practical considerations concerning the use of these tests. Nevertheless, more studies with placental histology as reference test are urgently required to reliably determine the accuracy of RDTs and PCR for the diagnosis of placental malaria. *P. vivax*-infections have been neglected in diagnostic test accuracy studies of malaria in pregnancy.

## Background

Malaria infection during pregnancy is a major public health problem in subtropical regions throughout the world. An estimated 125.2 million pregnancies occurred in areas with *Plasmodium falciparum *and/or *Plasmodium vivax *transmission in 2007, of which approximately 30.3 million occurred in Africa [1,2]. Of the five human malaria species, *P. falciparum *causes the most severe effects during pregnancy, and *P. vivax *is associated with maternal anaemia and low birth weight [3,4]. The effects of *Plasmodium malariae*, *Plasmodium ovale *and *Plasmodium knowlesi *infections in pregnancy are not well studied. *P. falciparum *infection during pregnancy is estimated to cause 10,000 maternal deaths each year and annually an estimated 75,000-200,000 infant deaths are linked to malaria in pregnancy (MiP) [1,2,5,6]. In low-transmission areas, *P. falciparum *infection during pregnancy usually presents as a symptomatic, severe disease that can result in death of mother and foetus. In high-transmission areas few *P. falciparum *infections result in fever and maternal death, but the newborn infant can be severely affected by intrauterine growth retardation and pre-term delivery [2]. Infants born after a pregnancy affected by malaria often suffer from anaemia and have an increased risk of contracting malaria themselves [2]. Furthermore, *P. falciparum *infection during pregnancy increases the risk of stillbirth [2]. The severe effects of *P. falciparum *and *P. vivax *malaria on pregnant women and their (unborn) infants make early detection and subsequent treatment of great importance. Even though there are control measures to prevent malaria infection during pregnancy, such as intermittent preventive treatment (IPTp) and bed nets (ITNs), diagnosis is essential in areas where there is anti-malarial or insecticide resistance. IPTp greatly reduces prevalence of malaria and severe consequences, but women are not protected throughout the entire pregnancy and can still become infected between doses or after the final dose, especially when other protective measures such as ITNs are not being used, or parasites are resistant to sulphadoxine-pyrimethamine. IPTp is mostly applied in areas where there is high malaria transmission, where women have acquired immunity and infection during the pregnancy is often asymptomatic, but not without consequences. Therefore, accurate diagnostic tools are necessary to confirm infection. Additionally, screening and subsequent treatment of women during pregnancy might be more effective than a preventive approach in areas with low levels of transmission or highly seasonal transmission [7,8]. Using IPTp in low transmission areas might result in a large proportion of pregnant women receiving sulphadoxine-pyrimethamine unnecessarily and a strategy has been proposed to screen pregnant women at antenatal care (ANC) visits with a rapid diagnostic test (RDT) and treat women who are positive with an effective anti-malarial [8]. An essential element of this strategy is good accuracy of the test and moreover it requires an affordable and quick diagnostic tool, such as an RDT.

*P. falciparum *infection in pregnant women presents differently than in non-pregnant women, where parasites are found in the circulation and can sequester to endothelial cells [9]. In pregnant women, *P. falciparum *malaria parasites express a different antigen variant (VAR2CSA) than in non-pregnant women, allowing them to sequester in the placenta and this is known as placental malaria [10]. Hence, in pregnant women peripheral parasitaemia can be absent or below the detection limit of microscopy [11,12].

While microscopic examination of blood slides is considered the 'gold standard' for diagnosis in non-pregnancy related malaria, accurate detection of parasite infection in the placenta requires examination of histological sections of fixed placental tissue [13-15]. An alternative is to examine placental blood with microscopy [11,12,15]. Placental histology and microscopic examination of placental blood can only be performed after delivery, when the placenta is available for examination. Since the detection of malaria parasites in the placenta is not possible during pregnancy, there is at this moment no other alternative than to detect the infection in peripheral blood.

There are alternatives to microscopic examination to test the peripheral blood though, such as rapid diagnostic tests (RDTs), which have the advantage of being quick and easy in remote settings. Depending on the manufacturer, the quality of the RDT in terms of accuracy and stability can be high [16,17]. RDTs are based on the detection of parasite antigens in the blood by specific monoclonal antibodies. RDTs for malaria detect one or more of the following antigens: Histidine Rich Protein 2 (HRP2), *Plasmodium *Lactate Dehydrogenase (pLDH) or Aldolase. In a recent systematic review comparing diagnostic accuracy of RDTs for uncomplicated *P. falciparum *infection, it was reported that HRP2-based RDTs have better sensitivity than pLDH-based tests, although specificity is better for pLDH-based tests [18]. In general, RDTs detecting HRP2 are most commonly used, because they are less expensive, more stable across a wider temperature range and have a lower detection threshold than pLDH-based tests [19,20]. HRP2-based tests, however, detect only *P. falciparum*, and antigenic variation of this antigen may cause false negative results [21]. The HRP2 antigen is excreted from the infected red blood cell, which can be beneficial for the diagnosis of placental malaria, as the antigen can be detected in the circulation even when the parasite is sequestered in the placenta [22].

Other alternatives for malaria diagnosis are DNA/RNA-based detection techniques, of which the polymerase chain reaction (PCR) is the most widely used [23,24]. PCR is considered to have the most sensitive detection level of parasites (for both regular peripheral malaria and placental malaria), but requires highly trained staff and specialized equipment, which are not always available in resource-poor settings [11,12,15].

Both PCR and RDTs are reported to have a higher sensitivity for placental malaria in peripheral and placental blood than microscopy, but are considered not to be as accurate as placental histopathology, however, evidence for this conclusion has not been summarized [11]. Besides accuracy there are many other reasons for choosing to use a certain type of diagnostic test, such as affordability, number of tests to be performed in a certain time, equipment, trained staff, etc., that depend on the setting and location in which the test will be used. Without a sufficient level of accuracy, however, there is no justification of using a certain test, even if it is practical and affordable and perhaps the only possibility in a certain situation. Therefore, the aim of the present study is to investigate the published diagnostic accuracy of RDTs and PCR for the diagnosis of malaria infection in pregnant women compared to a reference standard. These tests should at least have a better sensitivity and specificity than peripheral microscopy. Different consequences of the results will be discussed.

## Methods

### Eligible studies

Eligible studies were primary studies that assessed the diagnostic accuracy of RDTs, PCR, and microscopy of peripheral- or placental blood or placental histology for the detection of malaria in pregnant women. Studies included pregnant women (any age, gestation and parity) in malaria endemic regions (all human-infecting *Plasmodium *species). Studies that compared selected healthy controls to confirmed malaria patients (case-control) were not eligible for inclusion, because they tend to give an over-estimation of the sensitivity and specificity of the test under evaluation [25]. RDTs detecting any type of antigen (HRP2, pLDH, Aldolase), in any format (lateral flow cassette, dipstick, card etc.) and from any manufacturer were eligible as well as molecular diagnostic tests (PCR) in any format using *Plasmodium *DNA and/or RNA amplification.

### Definitions

Malaria infection of red blood cells in pregnant women can be found in both the peripheral and placental circulation and sequestered in the placenta and other organs or tissue. Placental malaria is defined as the presence of malaria parasites in placental tissue or blood in this study. Peripheral malaria infection is defined as the presence of malaria parasites in the circulation (peripheral blood). Both types of infection often occur at the same time and when it is not clear which of the two it is or specification is not desired, it is called malaria in pregnancy (MiP).

### Reference test: histology

Histological examination of a stained biopsy from the maternal side of the placenta is considered the gold standard for diagnosis of placental malaria. The biopsy is examined for the presence of malaria parasites and pigment in the placental tissue. Placental histology slides can be classified in active (parasites in the placenta), active chronic (parasites and pigment in placenta), past (only pigment in placenta) and no infection (no parasites or pigment in placenta) [13,14]. For the 2 × 2 tables both active and active-chronic infections were considered as positive for placental malaria and past and no infections as negative. Even though past infection is a clinically relevant outcome, and indicates that the participant has been infected during the pregnancy, for the purpose of comparing diagnostics only the current state of infection is of interest. In one study, past infection was considered as positive and the results were not presented for each class separately [26]. This study was not included in the meta-analysis, because it was the only study comparing RDT to histology. In two other studies, it was not specified if past infection was considered positive or negative. These studies were included in the meta-analysis and may be a source of bias [27,28].

### Reference tests: placental and peripheral blood microscopy

Histology is not available or practical in all situations, and not suitable for certain study designs. Instead, microscopic slide investigation of placental or peripheral blood is used as reference test. Slides are made from placental blood from the inter-villous space of the placenta, which can be collected in many different ways, for example, by aspiration with a syringe or as impression smear of placental tissue. For peripheral blood microscopy, venous or capillary blood is collected and thick and thin smears are made. Slides from blood of both sources are dried, fixed (thin smear), stained and examined microscopically for the presence of malaria parasites. Although peripheral parasitaemia during pregnancy has been related to placental infection at delivery, microscopy of peripheral blood indicates a different situation than histology; i.e. it detects parasites in the circulation (with or without placental infection) [29]. In order to gain more insight into the value of these different reference standards, they were evaluated against placental histology.

### Search strategy

Electronic databases were searched with the provided search terms. To avoid missing studies, the search terms were kept broad. The searches were performed in September 2009 in duplicate and updated in October 2010 and June 2011.

Medline (through Pubmed) was searched with ("malaria"[MeSH Terms] OR *Plasmodium *[All Fields] OR "malaria"[All Fields]) AND ("pregnancy"[MeSH Terms] OR "pregnancy"[All Fields] OR pregnan*[ti]).

EMBASE (through OVID) was searched with search terms: ('malaria'/exp or 'malaria'.mp. or ('plasmodium'/exp or 'plasmo*'.mp.)) and ('pregnancy'/exp or 'pregnan*'.mp.) and 'paludisme'.

In Web of Science the following search term was used: TS = ((malaria OR plasmo*) AND pregnan*).

The WHO library database (through e-library OPAC on WHO website) was searched with three terms 'malaria AND pregnancy', 'paludisme AND enceinte' and 'paludisme AND grossesse'.

Reference lists of the selected studies, narrative and systematic reviews and primary studies on malaria in pregnancy were manually checked for other relevant studies. Conference programmes and abstracts of recent conferences on malaria were consulted for recently conducted studies and the websites of the Roll Back Malaria programme, WHO and TDR were visited and searched for reports or publications [30-32].

### Selection of studies

A primary selection, based on title and abstract (compiled in reference manager [33]), was performed independently by two authors (JK and EO). Duplicate studies and studies that were not using diagnostic tests for malaria in pregnant women were removed. All studies considered relevant by at least one of the two authors were selected. If the full paper could not be retrieved online or through the central catalogue of Dutch academic libraries (NCC), and if the contact details could be retrieved, the authors were approached. In case the full paper was obtained, the same two authors independently assessed the study for inclusion, based on eligibility (described earlier) and the availability of data to derive 2 × 2 tables. Disagreements were resolved by discussion or by consulting a third author (PM or ML). Study information and fulfilment to the inclusion criteria was collected using an Epidata entry form [34]. If data for RDT evaluations were only partially reported, authors were contacted to provide the data if the contact details were available.

### Data extraction and management

Two authors (JK and EO) collected the data from included studies independently on forms prepared in separate Access databases (Microsoft 2003). Information about the study (title, authors, journal, etc.), study population and study design was collected, as well as descriptions of reference and index tests and data for 2 × 2 tables. The database was accompanied by a background document that explained how each item in the database should be interpreted and entered. After entry of all studies, the two databases were compared, and disagreements were resolved by discussion between the two data-collectors or if necessary with a third author (PM). Most disagreements in the two data sets turned out to be data entry errors.

Methodological quality was assessed using the QUADAS tool [35]. A study was considered to have a high risk of partial verification bias if more than 10% of the patients who received the index test did not receive verification of their true disease state, and the selection of patients to receive the reference standard was not random. A study was considered to have a high risk of differential verification bias if more than 10% of patients received verification with a different reference standard. Studies with a high risk of partial or differential verification bias were not included in the meta-analysis. A few additional items were added to the QUADAS tool that can be important for RDT accuracy. These items are: '*Has care been taken to store the tests at recommended circumstances (time, temperature, humidity)*?' and '*Was the staff that executed the reference standard trained to use this test?*'. In several studies more than two tests were compared, and multiple 2 × 2 tables could be extracted. Several quality items were considered for the separate test comparisons within one study.

### Statistical analysis and data synthesis

For each 2 × 2 table, the estimates of sensitivity and specificity and their 95% confidence interval were plotted in forest plots and receiver operating characteristic (ROC) space in Review Manager [36]. For the meta-analysis, metandi was used in Stata [37,38]. To perform metandi, a minimum of four 2 × 2 tables is required.

### Investigation of sources of heterogeneity

Diagnostic accuracy studies are expected to show considerable heterogeneity and the models used are by default random effects models, taking into account the between study variation as well as chance variation [39]. To further investigate the sources of heterogeneity, subgroup analyses was performed rather than including covariates in the meta-regression models, because metandi is not capable of including covariates in the analyses. The type of antigen (HRP2 or pLDH or Aldolase or in combination) detected by the RDT was investigated as a source of heterogeneity. For other supposed sources that might affect the accuracy of the RDTs, such as malaria species, gravidity, anti-malarial treatment and RDT brand, insufficient data was reported to determine if these issues affected accuracy.

## Results

### Results of the search

**Figure 1:**
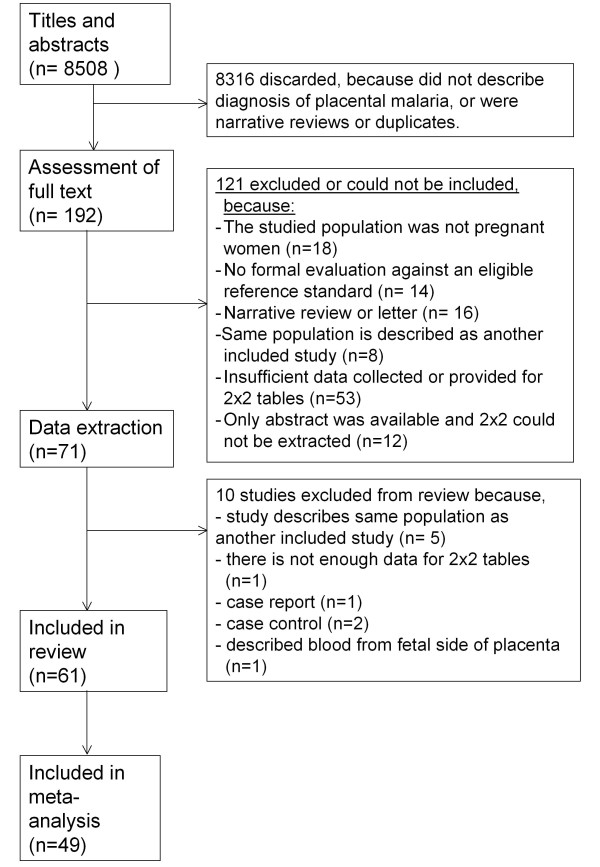
**Flow diagram of selection procedure**.

The searches in MEDLINE, EMBASE and Web of Science (WOS) retrieved 3,069, 3,167 and 2,249 studies, respectively. After removing duplicates, 169 studies were selected based on title and abstract. Additional searches retrieved another 23 studies. From these 192 studies, 131 were excluded for several reasons: full text paper could not be retrieved (n = 12), no pregnant women described (n = 18), insufficient data collected or provided for 2 × 2 tables (n = 54), narrative reviews or editorial or letter (n = 16), description of the same population as another study (n = 13), no formal evaluation against an eligible reference standard (n = 14) and various other reasons (n = 4) (Figure 1).

Additional file 1 lists the characteristics of the 61 included studies in the review [7,26,27,29,40-94]. Studies were performed between 1914 and 2009, and included approximately 45,000 women during pregnancy or at delivery. Most studies were conducted in sub-Saharan Africa (n = 52). Only four were conducted in South-East Asia (three in India, one in Thailand), and two in Yemen and one each in, Colombia, Panama and Papua New Guinea. Corresponding with the geographical locations, the majority of studies described *P. falciparum *infections (53 of 61 reported *P. falciparum *infection; in eight studies species were not specified). Only six studies reported *P. vivax *infections. *P. malariae *(n = 16) and *P. ovale *(n = 6) infections are rarely seen in the placenta, and were more often reported in peripheral blood as a mixed infection with *P. falciparum *and only in a small portion of patients (Additional file 1). There are not enough studies to perform subgroup analysis for the different species to determine if there is a difference in test accuracy to detect different malaria species, and most importantly between *P. vivax *and *P. falciparum*. Only 13 of 61 studies used placental histology.

### Methodological quality of included studies

**Figure 2:**
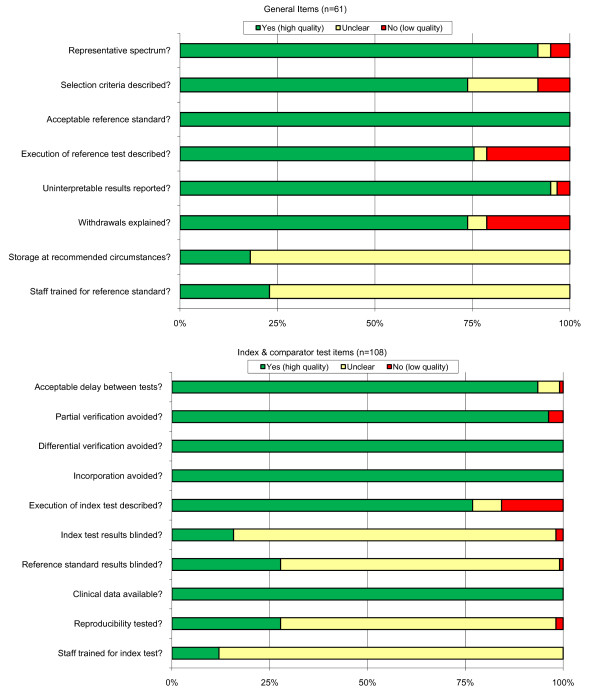
**Methodological quality assessment of all 61 included studies**. Top: general QUADAS items, scored for each study; Bottom: Items assessed separately for each index and comparator tests (n = 108) within the studies. Data presented as stacked bars representing the percentage of studies scored as 'yes' (green), 'unclear' (yellow) or 'no' (red) by the authors on each particular quality item.

The results of the quality assessment are presented in Figure 2. Most studies included a representative patient spectrum, but in two studies they included either only patients that were positive in a reference test or those that were negative [52,59]. Additionally, one study included patients that were positive in the index test and 30 negatives, but not randomly chosen [70]. Selection criteria were not sufficiently described in 16 studies [27,28,40,43,45-47,55,62,68,69,72,76,80,81,87]. Very little was reported on storage conditions of the tests and whether or not staff was trained for the reference or index tests. In about a quarter of the studies (n = 15) withdrawals were not explained [27,42,48,49,51,53,56,61,62,64,69,73,74,80,84].

Partial verification was a problem in four test comparisons (in four different studies), and these studies were removed from the meta-analysis [61,67,80,83]. Differential verification was not a problem in any of the comparisons. For most of the tests (n = 83) the execution of the test was described sufficiently, but in only a few comparisons (n = 17 for index test; n = 30 for the reference test) did the authors clearly mention that the interpretation of the tests was blinded, and only in a small number (n = 30) of the studies they reported that the reproducibility of the index test was tested.

### Findings

**Figure 3:**
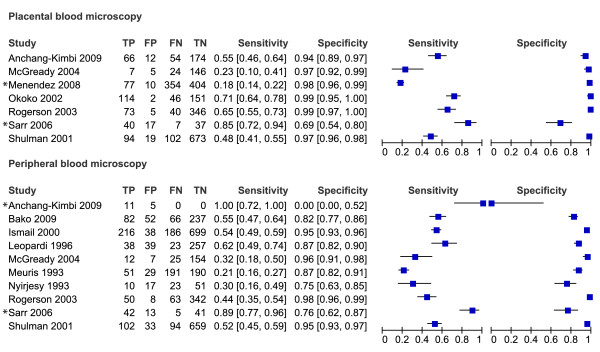
**Forest plots of sensitivity and specificity of microscopy of peripheral and placental blood with placental histology as reference test**. The squares represent the calculated specificity and sensitivity of one test within a study; the black line is the 95% confidence interval. Tests with a * in front were excluded from the meta-analysis, because of high risk of bias or complete data was not available.

**Figure 4:**
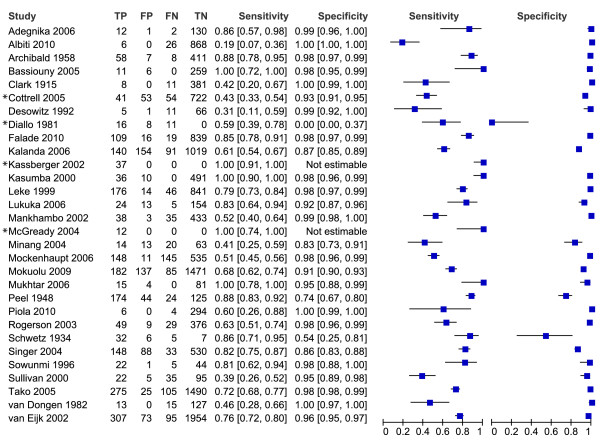
**Forest plots of sensitivity and specificity of peripheral blood microscopy with placental blood microscopy as reference test**. The squares represent the calculated specificity and sensitivity of one test within a study; the black line is the 95% confidence interval. Tests with a * in front were excluded from the meta-analysis, because of high risk of bias or complete data was not available.

**Figure 5:**
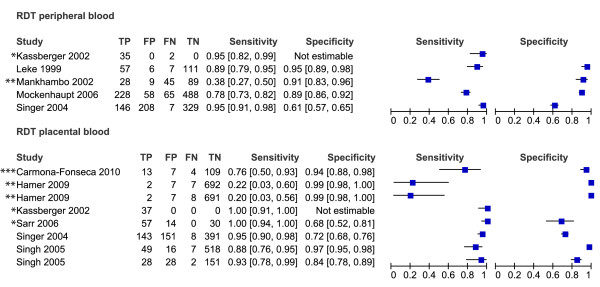
**Forest plots of sensitivity and specificity of RDTs with microscopy of placental blood as reference test**. The squares represent the calculated specificity and sensitivity of one test within a study; the black line is the 95% confidence interval. Tests with a * in front were excluded from the meta-analysis, because of high risk of bias or complete data was not available; with ** means that a pLDH-based RDT has been used; *** means that a HRP2-Aldolase-based RDT has been used.

**Figure 6:**
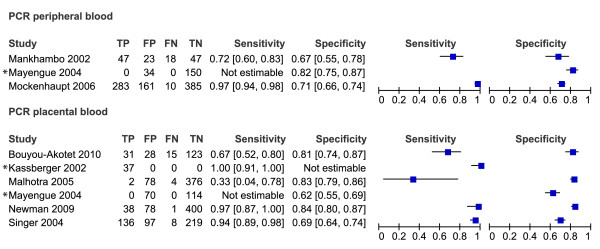
**Forest plots of sensitivity and specificity of PCR with microscopy of placental blood as reference test**. The squares represent the calculated specificity and sensitivity of one test within a study; the black line is the 95% confidence interval. Tests with a * in front were excluded from the meta-analysis, because of high risk of bias or complete data was not available.

**Figure 7:**
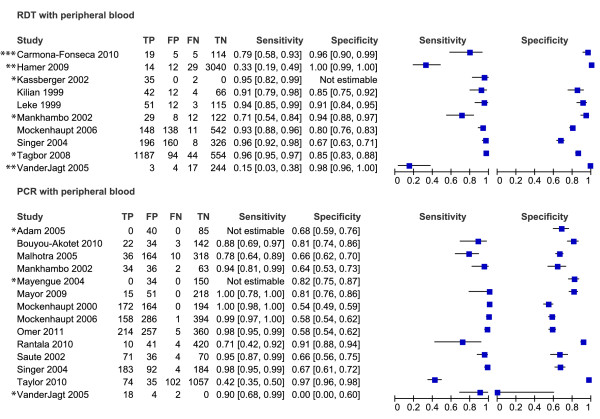
**Forest plots of sensitivity and specificity of RDTs and PCR with microscopy of peripheral blood as reference test**. The squares represent the calculated specificity and sensitivity of one test within a study; the black line is the 95% confidence interval. Tests with a * in front were excluded from the meta-analysis, because of high risk of bias or complete data was not available; with ** means that a pLDH-based RDT has been used; *** means that a HRP2-Aldolase-based RDT has been used.

Of the 61 studies included in the review, only 49 could be evaluated in the meta-analyses. These 49 studies are presented in the forest plots (Figures 3, 4, 5, 6 and 7). Some of the studies were not included in the meta-analysis, not enough studies to perform meta-analysis of these particular test comparisons were described and these studies are listed in Additional file 2 with the retrieved sensitivity and specificity. A substantial number of studies had to be excluded from final analyses, because there was not enough data to fill the 2 × 2 tables (n = 4) or because they suffered from partial verification bias (n = 2), or the 2 × 2 tables were only available for a subset of the patients (n = 3). In separate cases the studies were excluded, because the subgroups ended up being too small (n = 1), there was a too long a time delay between the tests (n = 1), or they used only matched negative cases (n = 1). The exact reasons for exclusion of each study are explained in the sections below. Only the most correctly-conducted studies were used to determine the accuracy and, therefore, the studies described above were excluded. The effect of exclusion of these studies was systematically examined, and in most cases the summary estimates were essentially unchanged. When the summary estimates were different when these tests were included in the analysis, this is discussed in the sections below.

The median prevalence of malaria (mostly *P. falciparum*) found by placental histology was 33.2% (range 17.2-52.5%). This median prevalence was retrieved from all included studies using histology and these studies were published between 1993 and 2009. These studies all included regular pregnant women presenting for delivery or recruited at ANC and followed till delivery [7,26,28,42,51,55,67,69,73,86].

### Peripheral and placental blood microscopy *vs *reference standard histology

**Figure 8:**
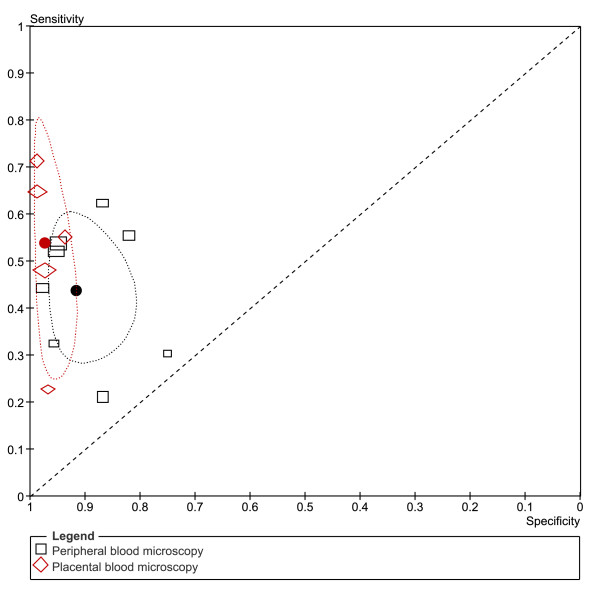
**Summary ROC plot of sensitivity and specificity of peripheral and placental blood microscopy with placental histology as a reference test**. The sensitivity of a test is plotted against 1-specificity, allowing comparison of both parameters at the same time for multiple tests. The rectangles and diamonds represent individual studies and size of the rectangles/diamonds is proportional to the number of patients included in the study. The thick round spots are the summary estimates of sensitivity and specificity and the dotted ellipses around the spots represent the 95% confidence intervals around the summary estimates. Black: peripheral blood microscopy; Red: placental blood microscopy. The reference test used to determine the plotted accuracies in this figure is placental histology.

In order to determine whether tests other than placental histology can be used as substitute reference standards, the accuracies of microscopy of placental and peripheral blood were evaluated. Seven studies evaluated microscopy of placental blood with placental histology as reference test, but one was excluded from the meta-analysis because there was a high risk of partial verification bias (Figure 3) [61,70]. Another study was excluded from the meta-analysis because it included women that were RDT positive and matched negative women [70]. The summary estimates of the five included studies in the meta-analysis for sensitivity and specificity were 54% [40-67 CI] and 97% [95-98 CI], respectively (Table 1 and Figure 8). Since the pathology of *P. vivax *infections during pregnancy has not been fully elucidated, the sensitivity and specificity of placental tests might be different for *P. vivax *infections compared to *P. falciparum*. If the study with *P. vivax *infections was excluded from the meta-analysis, there was a slight increase in sensitivity (60% [50-69 CI]) [7].

**Table 1:** Summary of findings

What is the diagnostic accuracy of microscopy of peripheral and placental blood to correctly identify histologically confirmed placental malaria (PM)?					
Population	Pregnant women				
Settings	At delivery where both placental and peripheral material is available; mostly *P. falciparum *infections				
Index test	Microscopic examination of placental or peripheral blood slide				
Reference Test	Histological examination of placental biopsies				

Type of test	Effect [95% CI]	Participants (studies)	Median prevalence (range)	Implications of results	Quality and comments

Microscopy of placental blood	Sensitivity 54% [40-67] Specificity 97% [95-98]	2153 (5)	24.4% (18.4-35.5)	With a prevalence of 25%, 25 out of 100 pregnant women will develop PM; 12 and 14 patients will be missed by placental and peripheral microscopy.	Representative patient spectrum; uncertain if all tests blinded; withdrawals poorly Reported.

Microscopy of peripheral blood	Sensitivity 44% [34-54] Specificity 92% [86-95]	4044 (8)	28.6% (17.2-52.5)	With a prevalence of 25%, 6 patients will be false positive in peripheral microscopy, but treatment is not harmful and can act as prophylaxis during the rest of the pregnancy.	Representative patient spectrum; uncertain if all tests blinded; withdrawals poorly reported; 1 study did not report selection criteri; 1 did not report the execution of the reference test; risk of verification bias in study.

**What is the diagnostic accuracy of RDTs and PCR to correctly identify PM confirmed by microscopy of placental blood?**					

Population	Pregnant women				
Settings	At delivery where both placental- and peripheral blood is available; mostly *P. falciparum *infections				
Index test	RDT or PCR with peripheral or placental blood				
Reference Test	Microscopic examination of placental blood slide				

Type of test/subgroups	Effect [95% CI]	Participants (studies)	Median prevalence (range)	Implications of results	Quality and comments

Microscopy of peripheral blood	Sensitivity 72% [62-80] Specificity 98% [95-99]	16609 (26)	15.9% (3.3-74.0)	Of 16 of 100 patients positive in placental blood, 4 would be missed in peripheral blood by microscopy.	Representative patient spectrum; 4 did not describe selection criteria; execution of index/reference test not reported in 13 tests

RDT of placental and peripheral blood					
Peripheral and placental blood pooled together	Sensitivity 81% [62-92] Specificity 94% [76-99]	3141 (5)	16.2% (2.4-11 34.9)	Of 16 of 100 patients positive with placental blood microscopy, 3 would be missed in any type RDT. Of 11 of 100 patients positive with placental blood microscopy, 3 patients would be missed by RDTs with placental blood. With a prevalence of 16%, 5 patients will be false positive with RDTs compared to placental blood microscopy.	Representative patient spectrum; uncertain if all tests blinded; withdrawals and uninterpretable results poorly reported; execution of test not reported in 3/7 tests; 1 study did not report selection criteria.
only placental Blood	Sensitivity 76% [44-92] Specificity 95% [87-99]	2124 (4)	11.20% (2.4 -22.6)		

PCR of placental and peripheral blood					
all types of PCR	Sensitivity 86% [65-95] Specificity 77% [71-82]	2608 (6)	18.5% (1.7-34.9)	Of 18 of 100 patients that test positive in placental blood microscopy, 3 would be missed by PCR, but 19 would be false positive.	Representative patient spectrum; uncertain if all tests blinded; withdrawals and uninterpretable results poorly reported.

**What is the diagnostic accuracy of RDTs and PCR to correctly identify microscopically confirmed peripheral malaria infection during pregnancy?**					

Population	Pregnant women				
Settings	During pregnancy, placental examination not possible; mostly *P. falciparum *infections				
Index test	RDT or PCR with peripheral blood				
Reference Test	microscopic examination of peripheral blood slide				

Type of test/subgroups	Effect [95% CI]	Participants (studies)	Median prevalence (range)	Implications of results	Quality and comments

RDT of peripheral blood					
all types (pLDH and HRP2)	Sensitivity 81% [55-93] Specificity 94% [82-98]	5340 (7)	17.60% (1.3-51.3)	Of 18 of 100 patients positive in peripheral blood microscopy, 3 would be missed in any type RDT. Of 28 of 100 patients positive in peripheral blood microscopy, 2 patients would be missed in HRP2 RDTs.	Representative patient spectrum; uncertain if all tests blinded; withdrawals and uninterpretable results poorly reported; test execution not reported in 3/7 tests; 1 study did not report selection criteria.
only HRP2 based	Sensitivity 94% [91-96] Specificity 81% [71-88]	1834(4)	28.10% (17.6-51.3)	With a prevalence of 28%, 14 patients will be false positive with HRP2 RDTs compared to peripheral blood microscopy.	

PCR of peripheral blood					
all types of PCR	Sensitivity 94% [86-84]	5741 (11)	19.0% (5.3-51.3)	Of 19 of 100 patients that test positive in peripheral blood microscopy, only 1 would be missed by PCR, but 20 would be false positive compared to peripheral blood microscopy.	Representative patient spectrum; uncertain if all tests blinded; withdrawals and uninterpretable results poorly reported;

From the ten studies that evaluated peripheral blood microscopy against placental histology, two were excluded from the meta-analysis. One study did not report false- and true negatives [42], and one study included women that were RDT positive and matched negative women [70] (Figure 3). The summary estimates for sensitivity and specificity of the included studies were 44% [34-54 CI] and 92% [86-95 CI] respectively (Table 1). If the case-control study was included, the summary sensitivity would have been 49% [35-64 CI]. When performing the meta-analysis with *P. falciparum *studies only (*P. vivax *study [7] removed) the sensitivity and specificity hardly changed (45% [34-56 CI] and 91% [84-95 CI] respectively).

In Figure 8 the summary ROC curve presents the summary estimates for placental blood and peripheral blood microscopy compared to histology. Placental blood microscopy had a slightly higher sensitivity and specificity than peripheral microscopy (Figure 8). There is, however, quite some overlap in the 95% confidence regions of the summary estimates, meaning that the true accuracy of the tests might be more alike (Figure 8). Both tests showed much variation in their sensitivity, ranging from 23% to 71% for placental blood and from 21% to 62% for peripheral blood, respectively. Neither of the two tests, however, reaches a summary sensitivity of at least 90%, and for peripheral blood microscopy the upper limit of the confidence interval is much lower than 90% specificity.

### Peripheral blood microscopy *vs *reference test placental blood microscopy

**Figure 9:**
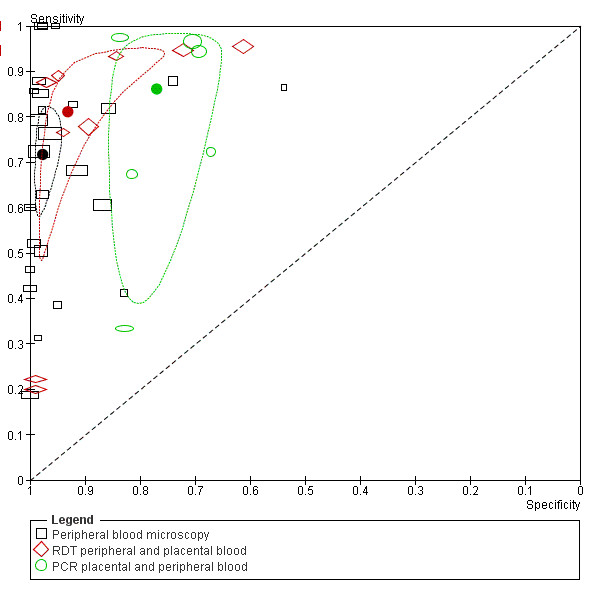
**Summary ROC plot of sensitivity and specificity of RDT and PCR of peripheral and placental blood and microscopy of peripheral blood with placental blood microscopy as reference test**. The sensitivity of a certain test is plotted against 1-specificity, allowing comparison of both parameters at the same time for multiple tests. The squares, diamonds and open circles represent individual studies and size of the rectangles/diamonds/circles is proportional to the number of patients included in the study. The thick round spots are the summary estimates of sensitivity and specificity and the dotted ellipses around the spots represent the 95% confidence intervals around the summary estimates. Black (squares): RDTs (detecting HRP2 or pLDH or HRP2-Aldolase). Red (diamonds): peripheral blood microscopy. Green (circles): PCR; The reference test used to determine the plotted accuracies in this figure is placental blood microscopy.

Many studies (n = 30) evaluated peripheral blood microscopy with placental blood microscopy as a reference, but four studies were excluded from the meta-analysis. Two of these four studies presented incomplete data for the 2 × 2 table [7,52], another only presented the 2 × 2 table for a subset of the patients (those with newborns with malaria) [48] and in the last study the delay between the sampling for the two tests is too long [29] (Figure 4). Sensitivity and specificity from the included studies are plotted in a summary ROC plot for peripheral blood microscopy with placental blood microscopy as reference test (Figure 9). The sensitivity varied from 19% to 100% with a summary estimate of 72% [62-80% CI] (Table 1 and Figure 9). The specificity, varied from 54% to 100%, and the summary estimate was 98% [95-99% CI] (Table 1 and Figure 9). There is one study where a *P. vivax *infection is observed in peripheral blood and not in placental blood, but the summary estimates are not different if this study is excluded from meta-analysis (sensitivity 73% [63-81 CI] and specificity 98% [95-99 CI]) [47].

### RDT and PCR *vs *reference test histology

The preferred reference standard for placental malaria remains histology, but unfortunately, only one study was included evaluating an RDT (HRP2-Aldolase) with peripheral blood against histology (sensitivity 57% [41-73 CI] and specificity 90% [80-96 CI]) [27]. One other study evaluated an RDT with placental blood to histology (sensitivity 100% [92-100 CI] and specificity 56% [41-69 CI]) [70]. No studies evaluated PCR against histology. Too few studies were collected for meta-analysis and therefore summary sensitivities and specificities of RDT and PCR and microscopy could not be compared.

### RDT and PCR *vs *reference test placental blood microscopy

As an alternative, some studies used microscopy of placental blood as a reference standard. Five studies compared RDT with peripheral blood to placental blood microscopy, and eight compared RDT with placental blood (Figure 5). Two studies were case-control studies, and were excluded from the meta-analysis [58,70]. One other study had incomplete 2 × 2 tables and the two 2 × 2 tables from this study were therefore excluded from the analysis [52]. This leaves two pLDH tests, one HRP2-Aldolase and six HRP2 tests with sensitivities varying from 78% to 95% for peripheral blood and 20% to 95% for placental blood. Not enough studies were available to pool the 2 × 2 tables RDTs of peripheral blood separately, but when RDTs of both peripheral as placental blood are pooled together, the summary sensitivity is 81% [62-92 CI] and summary specificity is 94% [76-99 CI] (Table 1 and Figure 9). If RDT of placental blood is pooled separately, similar sensitivity and specificity are found (Table 1).

Similarly, the 2 × 2 tables of PCR of peripheral and placental blood compared to placental blood microscopy can be pooled and analysed together. The 2 × 2 tables of two studies are not complete; therefore they were not included in the meta-analysis (Figure 6)[52,59]. Summary sensitivity (86% [65-95 CI]) is similar to RDT, but summary specificity (77% [71-82 CI]) is lower (Table 1 and Figure 9).

Compared to peripheral microscopy, RDTs and PCR have better sensitivity than when compared to placental blood microscopy as reference standard (Figure 9). RDTs and PCR, however, do have lower specificity, but based on the available data, it is not possible to conclude if these are indeed false positives, or infections that are missed by placental blood microscopy yet detected by RDT or PCR.

### RDT and PCR *vs *reference test peripheral blood microscopy

Although microscopy of peripheral blood is the least appropriate reference test for placental malaria as determined in a previous section, it has often been used as a reference test in practice. Many studies have been performed during pregnancy and not at delivery, which explains why peripheral blood microscopy is used. Ten studies compared RDTs (two pLDH and one HRP2-Aldolase and seven HRP2-based tests) to microscopy of peripheral blood and 14 compared PCR to peripheral microscopy. For both PCR and RDT, three studies each were excluded from meta-analysis because of incomplete data [40,52,59], case control [58] or high risk of verification bias [80,83] (only for PCR in [80]). Sensitivity and specificity is presented between quotation marks in the section below to underline the fact that peripheral blood microscopy is not an appropriate reference test for placental malaria.

**Figure 10:**
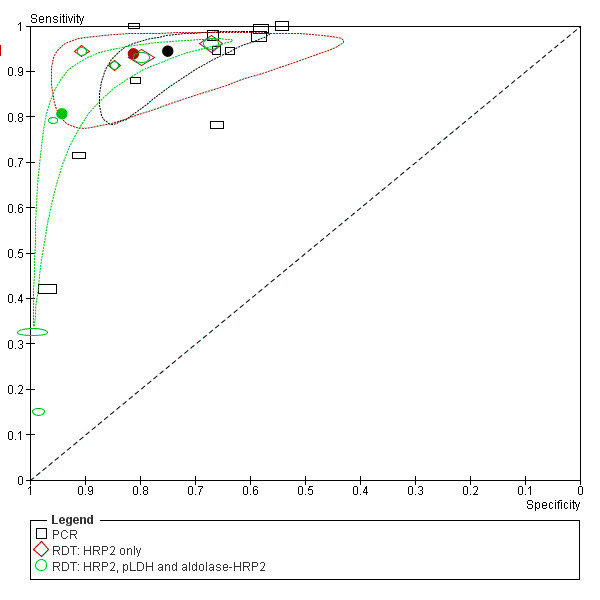
**Summary ROC plot of sensitivity and specificity of RDT and PCR with peripheral blood with peripheral blood microscopy as reference test**. The sensitivity of a certain test is plotted against 1-specificity, allowing comparison of both parameters at the same time for multiple tests. The rectangles, diamonds and circles represent individual studies and size of the rectangles/diamonds/circles is proportional to the number of patients included in the study. The thick round spots are the summary estimates of sensitivity and specificity for the different test types and the dotted ellipses around the spots represent the 95% confidence intervals around the summary estimates. Black (rectangles): PCR studies; Red (diamonds): studies with HRP2 based RDTs only; Green (circles): studies with RDTs, including both HRP2, pLDH and HRP2-Aldolase detecting tests. The reference test used to determine the plotted accuracies in this figure is placental histology. The inclusion of the pLDH and Aldolase-HRP2 based RDTs dramatically changes the summary estimate and confidence interval (green) compared to HRP2 tests alone (red), the sensitivity is much lower with the pLDH tests.

Analysis was performed for HRP2-based RDTs separately (Figure 7). Too few studies were retrieved in order to perform subgroup analysis on pLDH based RDTs, and therefore a sensitivity analysis was performed by comparing all RDT studies with a subgroup of HRP2-based RDTs. The summary estimate of "sensitivity" was higher for the HRP2 subgroup (94% [91-96 CI]) compared to the overall analysis for all RDT types (81% [55-93 CI]). For the summary estimate of the "specificity", however, the opposite was observed: 81% [71-88 CI] for the HRP2 subgroup and 94% [82-98 CI] for all type RDTs (Table 1 and Figure 10). Although the difference is not significant, it might indicate that, at least for placental malaria, there is a difference in accuracy between the different RDT types (Figure 10).

In studies where PCR was compared to microscopy of peripheral blood, a quite good "sensitivity" was found (71% - 100%) except for one outlier (42%), but a low "specificity" was observed, varying from 54% to 97% (Figure 7). This resulted in the summary estimates of "sensitivity" and "specificity" of 94% [86-98 CI] and 75% [63-84 CI] respectively (Table 1 and Figure 10).

## Discussion

To estimate the accuracy of RDTs for diagnosing malaria infection in pregnant women, the results of 49 studies were analysed. Few studies exist that fully evaluate microscopy, RDTs and PCR against the gold standard, placental histology, and each other. This makes it difficult to directly compare the accuracies of the different tests, and therefore currently no reliable data about the accuracy of RDTs and PCR for the detection of placental malaria is available. The present study shows that microscopy of both placental and peripheral blood do not detect many placental infections that are identified by histology (sensitivities of 54% and 44%) and cannot reliably replace histology as a reference standard for placental *P. falciparum *infection. Despite these limitations RDTs, especially HRP2-based tests, appear to have reasonable accuracy compared to microscopy. The World Health Organization (WHO) together with the Foundation for Innovative New Diagnostics (FIND) have performed extensive testing of many RDT devices and there is a great need to compare the best tests from that evaluation with histology, microscopy and PCR in pregnant women [20].

When using placental microscopy as a reference test, PCR has the best sensitivity, followed by RDT and both have higher sensitivity than peripheral microscopy. Peripheral microscopy, however, has the highest specificity, followed closely by RDT. Based on these results, RDTs seem a good alternative for diagnosis of placental malaria. For the determination of the accuracy of RDT and PCR compared to placental blood microscopy, however, tests performed on both peripheral and placental blood were pooled. This might have introduced bias, since the starting material is not the same, and therefore, the accuracy might be different. There were, however, too few studies performed on peripheral blood alone to perform meta-analysis. The pooled summary sensitivities and specificities are not that different from the summary values of these tests using placental blood, but this may be due to the fact that these tests are a large proportion of the pooled analysis.

Compared to the imperfect reference standard peripheral microscopy, the proportion of microscopy positives detected by any RDT ("sensitivity") was 81% [51-95 CI]. The proportion microscopy negatives, with a negative RDT ("specificity"), was 94% [76-99 CI]. As the RDTs seem to miss patients that are positive in microscopy, this is not very reassuring for the value of RDTs for the diagnosis of malaria in pregnant women. Nevertheless, HRP2-based RDTs might have adequate sensitivity (94% [91-96 CI]).

The results in this study suggest that the proportion of microscopy positives detected by HRP2-based RDTs compared to peripheral microscopy is higher than for pLDH-based RDTs. The proportion microscopy negatives with a negative RDT, however, is lower for HRP2 RDTs compared to pLDH RDTs. This pattern is similar to the results of a meta-analysis of RDTs for uncomplicated malaria, and the sensitivity of HRP2-based RDTs for pregnant women (94% [91-96 CI]) is very similar to that for uncomplicated malaria (95% [93-96 CI]) [18]. The specificity compared to peripheral microscopy for HRP2-based RDTs for pregnant women (81% [71-88 CI]), however, is much lower than for uncomplicated malaria (95% [93-99 CI]). A possible explanation for this observation is that peripheral microscopy is not a suitable reference test for placental malaria and does not detect all placental infections, whereas the HRP2 RDTs might be able to detect these infections, resulting in a lower specificity when compared to peripheral microscopy. This is strengthened by the specificity of HRP2 RDTs (90% [84-95 CI]) compared to placental blood microscopy, which detects more placental infections than peripheral microscopy. Direct comparisons of HRP2 RDTs with histology are needed to confirm this.

For PCR, the proportion of microscopy positives detected ("sensitivity") was 98% [91-99 CI] and the proportion microscopy negatives, with a negative PCR ("specificity"), was 65% [59-72 CI]. PCR may seem to miss fewer patients with peripheral *P. falciparum *parasites than an RDT, but does indicate a number of women without parasites detected by microscopy as having malaria. Whether these are cases that were missed by microscopy or whether these were false positive PCR results, resulting in low specificity, needs to be further investigated.

The sensitivity and specificity of tests vary with parasite density, and many included studies report lower sensitivities at lower parasite densities (two studies for PCR and RDT; seven for RDTs; four for microscopy). This factor is a particular challenge for malaria infections during pregnancy in both high and low transmission settings, especially for multigravid women who are often reported to have lower parasite densities. Of the included studies, 13 report higher parasite densities in peripheral and/or placental blood for primi- and/or secundigravidae. In high transmission areas, women have acquired immunity during their life, and although they might have substantial placental sequestration (especially in primi- and secundigravidae), they can have a lower amount of parasites in the circulation by clearance of infected red blood cells by the immune system. In low transmission areas, women have low or no immunity and women can get sick at initially low parasite densities. Therefore it is very important for the diagnosis of malaria in pregnant women that the test has sufficient accuracy at lower parasite densities, and low parasite density might partially explain the poor performance of pLDH RDTs.

In a previous report, prevalence of placental malaria estimated by different tests was compared within each study [11]. However, even if the prevalence detected by one test is higher than the prevalence detected with the other test, it does not necessarily mean that the same cases are detected. Additionally, in this way it is not clear whether the positives found by each test are true positives or false positives. Nevertheless, the conclusions of that report and this review are broadly similar. Histology found a higher prevalence than placental blood microscopy and placental blood microscopy in turn found a higher prevalence than peripheral microscopy in four out of six studies. In both peripheral and placental blood, in nearly all studies, higher prevalence was found using PCR than using RDT and higher prevalence was found using RDT than using microscopy. No studies compared prevalence estimated by RDT and PCR to prevalence found by histology.

The results presented in this review are mainly applicable to sub-Saharan Africa, as 85% of the included studies were conducted in that area. It is surprising that only a few studies were available evaluating diagnostics in the Asian-Pacific region, considering that most pregnancies at risk in the world are located in this region [6]. In line with this observation, most studies described *P. falciparum *infections, and only six studies reported *P. vivax *infections. Meta-analysis was based on studies of which the majority described *P. falciparum *infections and studies with *P. vivax *infections had little influence on the outcome. Therefore, conclusions in this review are mainly applicable to *P. falciparum *infections. Due to the potential pathological differences between *P. falciparum *and *P. vivax *infection in pregnancy a difference in diagnostic accuracy of the various tests is expected between the species and more studies should aim at evaluating diagnostics in *P. vivax*-infected pregnant women in the future. Too few studies with *P. vivax *infections were included in the meta-analysis to be able to determine if there is a difference in accuracy.

The use of PCR for diagnosis of (placental) malaria remains a matter of discussion, especially in the submicroscopic cases, because it is difficult to determine what the PCR is detecting. It is very sensitive in detecting parasite nucleic acids, but it is unclear if this is a residual from a non-viable sequestered parasite, or a viable parasite or gametocyte. Additionally, there is some discussion whether these low parasite levels detected by PCR are clinically relevant. Several studies have tried to address this issue by researching the association of a positive (submicroscopic) PCR result and outcome measures such as anaemia, low birth weight and premature delivery. A systematic review has been conducted to summarize this data and concluded that the frequency of anaemia is significantly lower in uninfected women compared to women with a submicroscopic infection, although the risk is lower than with microscopic infections, and a similar pattern is found for low birth weight [95]. The review on the effects of submicroscopic infections did not include three studies that have been described in the current study [60,90,93]. One of these three studies described a significantly increased risk of anaemia with submicroscopic infections compared to PCR- and microscopy negative women, as well [60]. One of the other studies describes that submicroscopic infection was predictive of low birth weight in HIV positive, but not HIV negative women [90]. More comparisons with histology might shed more light on this issue and show that a significant proportion of the peripheral submicroscopic infections, are in fact placental infections. As mentioned before, pregnant women often have low parasite densities, and tests should have good sensitivity at these low densities; PCR techniques often have better sensitivities at low parasite densities than RDTs and microscopy.

## Conclusions

Currently, there is no reliable data about the accuracy of RDTs and PCR for the detection of placental malaria. This is because the studies done so far used invalid reference standards. Before a firm conclusion can be drawn about whether RDTs or PCR can be used to detect placental malaria, these tests should be evaluated against histological examination of the placenta. Direct comparisons of RDTs and PCR against histology *versus *peripheral microscopy against histology are needed to decide whether RDTs or PCR have better accuracy for placental malaria than (currently used) peripheral microscopy.

## List of abbreviations

ANC: antenatal care; CI: 95% confidence interval; HRP2: histidine rich protein II; IPTp: intermittent preventive treatment; ITNs: insecticide treated nets; MiP: malaria in pregnancy; NCC: Nederlandse centrale catalogues (Dutch central catalogue); PCR: polymerase chain reaction; pLDH: *Plasmodium *lactate hydrogenase; PM: placental malaria; RDTs: rapid diagnostic tests; ROC: receiver operating characteristic; SROC plot: summary receiver operating characteristic plot; TDR: UNICEF/UNDP/World Bank/WHO Special Programme for research and Training in Tropical Diseases; WOS: Web of Science; QUADAS: Quality Assessment of Diagnostic Accuracy Studies.

## Competing interests

The authors declare that they have no competing interests.

## Authors' contributions

JK, PM and HS conceived the idea for this study. JK, PM, KB and ML designed the study. JK and EO performed the searches for studies, reviewed the retrieved studies and made the primary selection of eligible studies. Inclusion of the studies and methodological quality was assessed by JK and EO, and disagreement were resolved with PM and/or ML. Data was collected by JK and EO, and analysed by JK and ML. JK prepared the first draft of the paper and all authors contributed to the writing of the report and have reviewed and approved the final version.

## Supplementary Material

Additional file 1 **Characteristics of included studies**. All studies included in the review are listed in this table. The clinical features, study design and participants are given for each study individually. The reference and index tests are listed that were used in each study and the prevalence as measured by the reference test is reported together with the encountered malaria species. The notes section contains comments on possible bias that may be introduced by that study and prevalences determined by the index tests, if reported. Click here for file

Additional file 2 **Evaluations not included in the meta-analysis**. This file lists the evaluations that were not included in the meta-analysis or discussed in the review, because of the low number of studies, or use of reference test. The sensitivity and specificity and their 95% confidence intervals (CI) are listed for each comparison of index and reference test for each study if it was possible to extract all data. Click here for file
